# Design considerations for hypertension chronotherapy trials: insights from experience and modelling

**DOI:** 10.1186/s12916-026-04915-8

**Published:** 2026-05-19

**Authors:** Olivia Walch, Amy Rogers, Yitong P. Huang, Marc D. Ruben, Kenneth A. Dyar, Robert W. V. Flynn, Isla S. Mackenzie, Roberto Manfredini, Francesco P. Cappuccio, Filippo Pigazzani

**Affiliations:** 1 Department of Neurology, University of Michigan, Ann Arbor, MI USA; 2 Arcascope Inc, Arlington, VA USA; 3 Division of Cardiovascular Research, School of Medicine, MEMO Research, University of Dundee, Dundee, UK; 4 Department of Mathematical Sciences, Smith College, Northampton, MA USA; 5 Division of Pulmonary Medicine and Biomedical Informatics, Cincinnati Children’s Hospital, Cincinnati, OH US; 6 Metabolic Physiology, Institute for Diabetes and Cancer, Helmholtz Diabetes Center, Helmholtz Munich, German Research Center for Environmental Health and German Center for Diabetes Research (DZD), Munich, Germany; 7 Department of Medical Sciences & University Strategic Center for Studies on Gender Medicine, University of Ferrara, Ferrara, Italy; 8 Warwick Medical School, Sleep Health & Society Programme, University of Warwick, Coventry, UK

**Keywords:** Chronotherapy, Chronotherapy Trial, Chronotype, Dosing time, Hypertension, Mathematical Modelling, Trial Design, Randomised Clinical Trials

## Abstract

Chronotherapy aims to maximise treatment efficacy while minimising side effects by scheduling treatment according to personal biological rhythms. In recent years, randomised clinical trials (RCTs) have been conducted to evaluate whether scheduled blood pressure interventions can improve patient outcomes. However, reports of time-of-day effects have attracted rebuttals and engendered methodological debate. A perfectly controlled chronotherapy trial (i.e., a trial that assesses the effect of assigning time of intervention) will never be feasible in the real world; yet some factors may be more critical to consider and control for than others. To advance the conversation about how best to evaluate the effects of antihypertensive chronotherapeutic interventions in RCTs, we discuss critical considerations for clinical trials of chronotherapy for hypertension and apply mathematical modelling to provide quantitative insights into the extent to which such factors may influence the detected effect size.

**Supplementary Information:**

The online version contains supplementary material available at 10.1186/s12916-026-04915-8.

## Background

More than half of human genes are rhythmically expressed with a period of approximately 24 h [1–3]. This circadian variation in gene expression reflects complex interactions between environmental cues (mainly light/dark cycles) and our endogenous timing system, which consists of ~ 24-hour cellular molecular clocks. The central circadian clock in the hypothalamic suprachiasmatic nucleus (SCN) coordinates rhythmic behaviours, including sleep-wake and fast-feed cycles. It thereby sustains homeostasis by synchronising peripheral tissue clocks that control rhythmic hormone levels, body temperature, metabolism, immune responses, and cardiovascular function [4]. 

Chronotherapy aims to use knowledge of circadian rhythms to maximise treatment benefits and minimise potential side effects and toxicity. Time-of-day-dependent treatment responses have been reported across many interventions and disease states [5–7]. However, these outcomes have not been consistently replicated [8–10]. 

In the case of hypertension, both the MAPEC (Ambulatory Blood Pressure Monitoring for Prediction of Cardiovascular Events) and the Hygia trials suggested that evening dosing of antihypertensives was associated with a large additional benefit on cardiovascular outcomes despite only modest reductions in 24-h blood pressure (BP) [7, 11]. However, these trials raised concerns regarding methodological transparency and potential biases [12, 13]. More recently, other trials indicated contrasting results. The pragmatic Treatment in Morning vs. Evening (TIME) study, BedMed, and BedMed-Frail chronotherapy trials, which assessed the effect of dosing time, found no significant difference in cardiovascular events between evening or morning dosing of antihypertensive drugs [10, 14, 15]. Hence, although some inconsistencies may have arisen from methodological differences, it remains unclear how to interpret these conflicting evidence.

We propose key considerations for designing hypertension chronotherapy trials to increase the likelihood of consistent results and to advance the field of chronotherapy. We used simple mathematical models to demonstrate how choices around what to control in randomised controlled trials (RCTs) of chronotherapy for hypertension should be determined by the individual trial’s goals, with some elements recommended to be accounted for in *all* chronotherapy trials. All simulations were carried out with Python 3.9.6, and all code is available at https://www.github.com/ojwalch/time-simulations.

We frame our discussion using the TIME study as an illustrative example. TIME was a large (21,104 participants) RCT that evaluated the effects of dosing time of antihypertensive drugs on cardiovascular outcomes in adults with hypertension [10]. As TIME found no difference in cardiovascular outcomes between morning and evening administration of blood pressure medications, we might conclude that chronotherapy for hypertension has no relevance in the prevention of cardiovascular disease in treated hypertensive patients. However, the Chronotype sub-study of TIME (with over 5,000 participants), which was designed to investigate possible interaction between the timing of antihypertensive medication and the patient’s chronotype (i.e., an individual’s preferred timing of sleep and activity, measured by questionnaire), found a reduced risk of non-fatal myocardial infarction when blood pressure treatment dosing was matched to the individual chronotype – i.e., personalised chronotherapy [16]. The findings of the BedMed chronotype sub-study recently supported similar conclusions [17]. 

These recent findings have reignited interest in chronotherapy for hypertension, making it more relevant than ever to explore how the design of chronotherapy trials may affect observed outcomes [18]. 

## Main text

### Variables to consider in hypertension chronotherapy trials

#### Dosing time history

Antihypertensive treatment history can influence the outcomes of patients with hypertension. Indeed, delaying the initiation or intensification of ongoing antihypertensive medication increases the risk of acute cardiovascular events, potentially due to an accumulating hypertensive-mediated organ damage [19–21]. 

In the TIME study, all participants had an equal chance of being assigned to either the morning or evening dosing time group, regardless of prior dosing experience. 85.4% of them had previously taken their antihypertensive drugs in the morning [10]. Although the length of time spent on any prior dosing schedules before study onset was not recorded, the mean age of participants was 67.7 years, suggesting that many had likely been taking antihypertensive medications for several years before enrolling in the trial.

*Would considering dosing time history have made a difference?* In the presence of an assumed therapeutic effect of dosing time, years of previous exposure to a sub-optimal time could bias the observed efficacy towards the null. For example, consider an individual who, for fifteen years before joining a trial, has been taking their medication in the morning. If this individual is randomly assigned to take their medication in the evening for a five-year trial, by the end of the trial, one-quarter of their lifetime medication exposure will have occurred at night, and the remaining three-quarters will have occurred in the morning. Where a trial endpoint has a long or unknown natural progression, attributing that event entirely to the dosing time during the trial could be misleading.

**Fig. 1:**
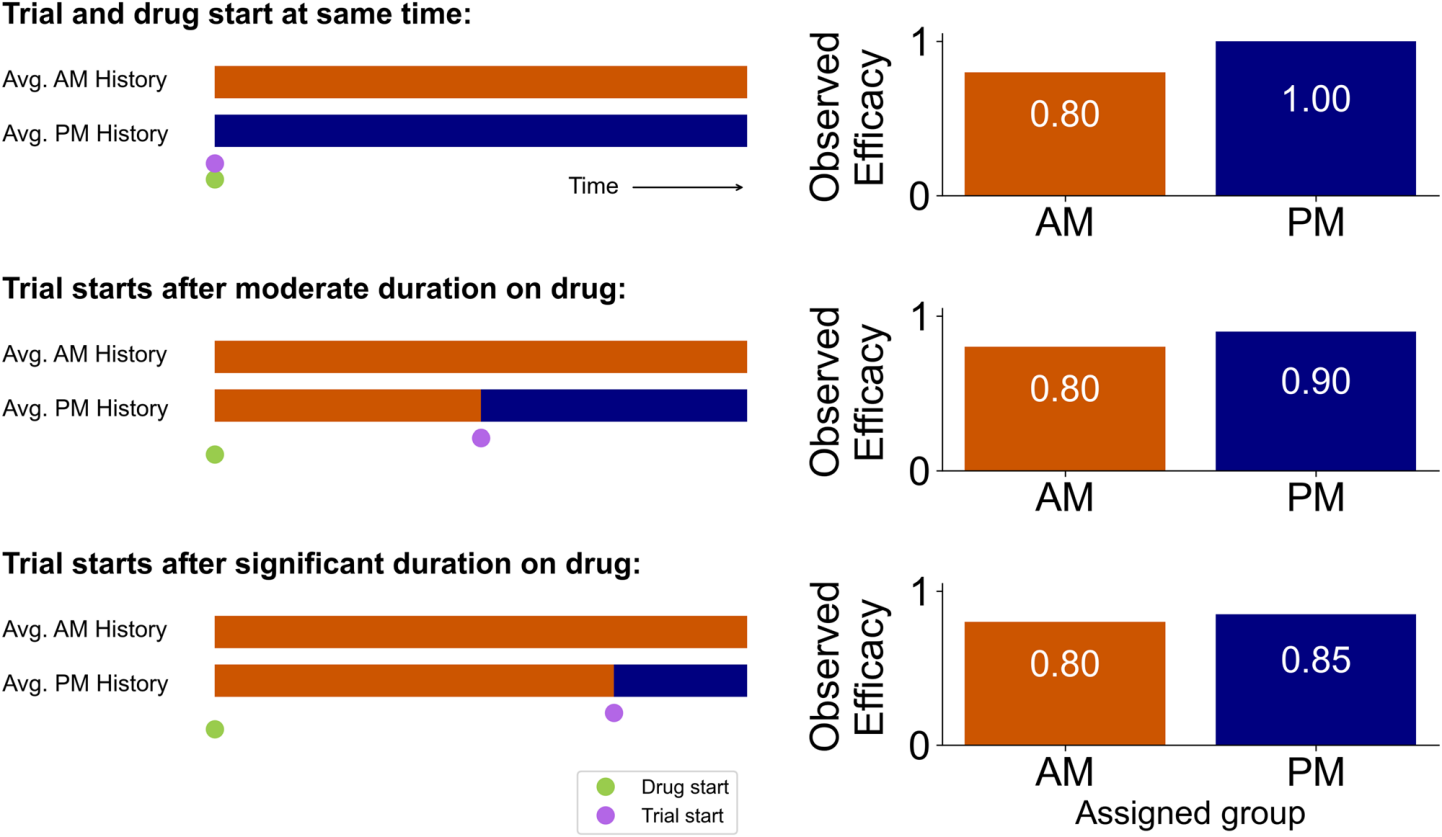
Hypothetical impact of prior dosing history on observed efficacy. A true 20% efficacy difference is assumed between a.m. and p.m. dosing (a.m. 20% worse). Left panels illustrate possible dosing histories, with orange bars indicating time on a.m. dosing and purple bars indicating time on p.m. dosing; drug start and trial start are marked with circles. The average participant is assumed to have dosed in the a.m. before trial enrolment, as in TIME. Right panels show observed efficacy, approximated as a weighted sum, with a.m. and p.m. efficacies weighted by how much time was spent by the average participant on that dosing schedule. As the duration of prior a.m. dosing increases, the apparent difference between randomised dosing groups decreases. For simplicity, this model assumes proportional effects and does not incorporate age-dependent risk, lag effects, or time-dependent nonadherence. The net efficacy is derived using the formula (0.8) * (0.75) + (1.0) * (0.25) = 0.85

To illustrate this, we constructed a simple model assuming a true 20% efficacy advantage of p.m. over a.m. dosing. In the model, overall efficacy for an ‘average’ participant is a weighted sum of the efficacy during years of prior a.m. dosing and efficacy during the trial dosing schedule. As the duration of prior a.m. dosing increases, the apparent difference between randomised a.m. and p.m. dosing groups is progressively attenuated, even though the true time-of-day effect is unchanged (Fig. 1). Under plausible assumptions about long pre-trial exposure to morning dosing, a 20% underlying benefit of evening dosing could appear much smaller in the trial data. It is important to note that more complicated models, such as ones that more heavily weight recent dosing time and incorporate saturating effects on overall efficacy, would blunt this effect, yet the general trend of reducing the observed differences between dosing time groups would persist. Given the potential bias introduced by prior dosing history, an ideal chronotherapy trial design could be to restrict participation to incident users, i.e., only those individuals who are starting to take the drug in question for the first time. However, this would severely limit recruitment.

For future hypertension chronotherapy trials that include prevalent medication users and where the effect of the intervention could be proportional to the exposure duration, we recommend asking participants to report the duration of prior antihypertensive drug exposure and their usual dosing schedule; this would allow the analysis to adjust for this potential confounder.

#### Variation in individual biological time

Substantial inter-individual variability has been observed in human circadian rhythmicity. Some individuals have intrinsic circadian periods that are shorter or longer than average [22]. These inherent biological rhythms influence the personal tendency towards specific sleep and wake times, i.e., “individual circadian preference,” also referred to as chronotype. Individuals with shorter circadian periods tend to wake earlier and may be referred to as “early chronotypes” or “morning larks”. Conversely, those with longer circadian periods may be categorised as “late chronotypes” or “night owls”, who tend to wake later and feel alert later in the evening or night [23, 24]. 

These inherited inclinations interact with external cues, including light and non-photic stimuli (e.g., meal timing, exercise, temperature), to determine an individual’s biological time (i.e., endogenous circadian phase in tissues/organs of interest) [25]. Unlike external clock time, “biological time” is personal; it can vary from day to day and is continually modulated by external cues. Due to inter-individual differences in circadian timekeeping (i.e., chronotype), as well as recent patterns of activity (e.g., long-haul trans-meridian travel, shift work), the same external clock time, such as 8 a.m., could represent very different biological states in two trial participants living in the same geographic location. Furthermore, the same individual could have a markedly different biological state on Monday versus Sunday, despite being assessed at 8 a.m. on both days. Additionally, chronotype has been linked to health outcomes, with those of late chronotype being more likely to experience cardio-metabolic dysfunctions and to be at greater risk of developing cardiovascular disease [26, 27]. 

Difficulties in targeting circadian time at the individual level have led most chronotherapy trials to evaluate more generalised time-of-day effects (i.e., morning dosing vs. evening dosing) rather than personalised interventions anchored to circadian biomarkers, such as “four hours before dim light melatonin onset (DLMO)”, the gold-standard circadian phase marker [10, 28]. 

Participants in the TIME study were advised to take their antihypertensive medications in one of two randomly assigned time windows: 6a.m. to 10 a.m. or 8 p.m. to midnight, i.e., the intervention was based on the external clock, or local time. The TIME study was not designed to measure the effect of biological dosing time (the interaction between drug administration and biological time).

This pragmatic approach, using external clock time instead of biological time, may explain some of the heterogeneity in the results of clinical trials evaluating chronotherapeutic interventions [12]. 

**Fig. 2:**
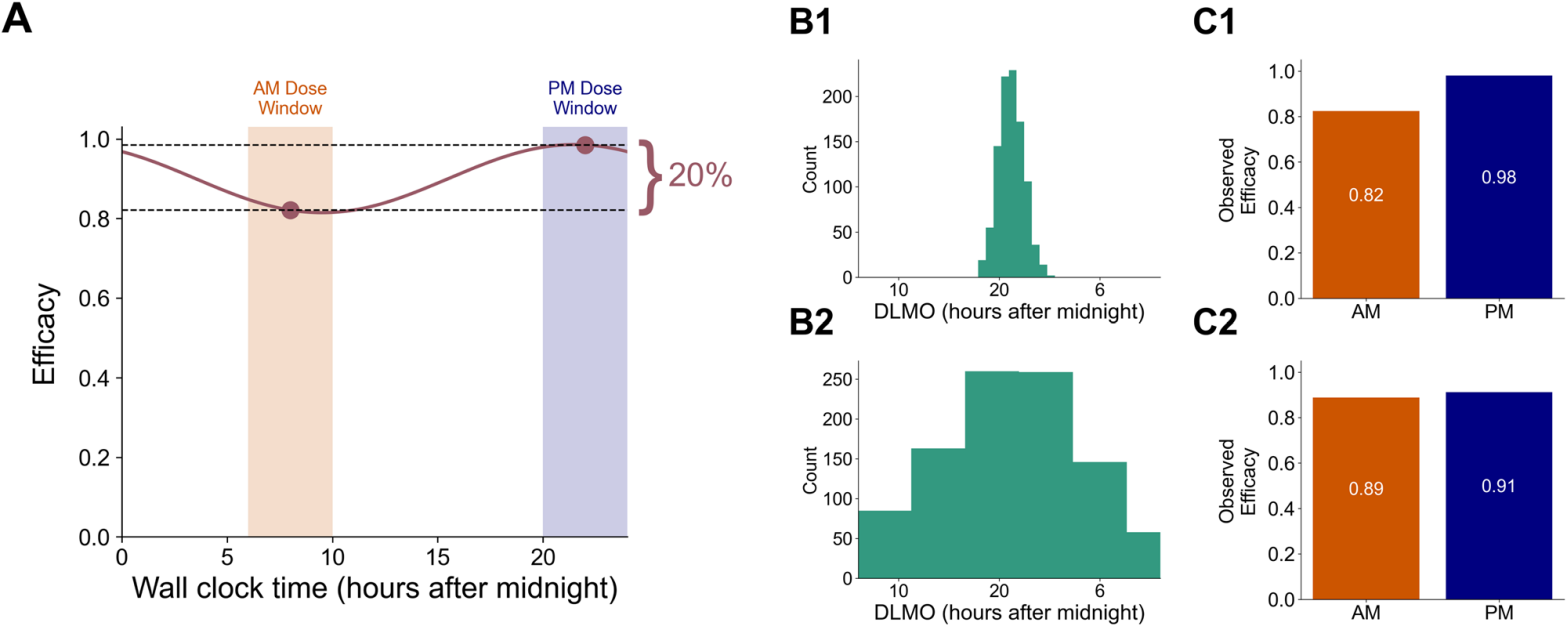
Effect of dosing strategy and population circadian distribution on observed effect size. **A** Hypothetical intervention efficacy curve with evening as the optimum dosing time, such that dosing at 0800 is 80% as effective as dosing at 2200 for the average person, in which dosing at 08:00 for the average person is 80% as effective as dosing at 22:00. **B1** Distribution of dim light melatonin onset (DLMO) from a relatively homogenous population of healthy adults (data from Cox et al. 2024; mean age = 26.7 years, SD = 7.2) [29]. **C1** Simulated observed efficacy for the DLMO distribution in (**B1**), obtained by sampling the efficacy curve in (**A**) at a.m. and p.m. clock times offset from the optimal times according to each individual’s DLMO difference from the mean (21.22 h after midnight). Under this narrow distribution, the observed a.m. - p.m. difference closely matches the true 20% efficacy difference. (**B2**) Wider DLMO distribution representing a more circadian-disrupted population (e.g., shift workers). **C2** Corresponding simulated observed efficacy for (**B2**) showing substantial attenuation of the a.m. - p.m. difference (a.m. efficacy is 97% of p.m. efficacy for the evening-optimal curve).

Figure 2 illustrates how the population distribution of biological time can modify the observed effect size between the a.m. and p.m. dosing. We simulated an intervention with a true 20% efficacy advantage for evening dosing and then sampled this efficacy curve using either a narrow distribution of biological time (similar to a healthy young adult cohort) or a broader distribution (as might be seen in shift workers). With a narrow distribution, dosing by external clock time closely preserves the true a.m. - p.m. difference, because most individual biological time is aligned with the average. In contrast, with a broader distribution, misalignment between biological time and external clock time substantially attenuated the apparent difference between a.m. and p.m. dosing, despite the underlying true effect being unchanged [29, 30]. This analysis implies that the extent to which chronotype can introduce a confounding effect depends on the heterogeneity of the participant population and the shape of the intervention efficacy curve. A highly homogeneous population, where biological times are concentrated in a narrow range (such as a healthy population with a narrow age range), may not require personalisation for a chronotherapeutic effect to be observed. However, a heterogeneous population may require more accurate proxies for circadian time than sleep and wake, such as wearable actigraphy tracking or moderately invasive biological sampling (e.g., salivary melatonin assays, urinary 6-sulfatoxymelatonin, plasma melatonin and cortisol levels, or core body temperature) [30–32]. While we are unaware of any studies quantifying the distribution of DLMO values for hypertensive patients, older individuals tend to have narrower and earlier distributions of DLMO than younger individuals, suggesting that the population in TIME may have more closely resembled the homogeneous population in our simulations than the disrupted population [33]. 

Yet, in a recent sub-cohort analysis of the TIME study using participant-reported chronotype information, the risk of hospitalisation for non-fatal myocardial infarction was higher for later chronotypes dosed in the morning and significantly lower for later chronotypes dosed in the evening [16]. Moreover, the same risk was lower for individuals with earlier chronotypes who were dosed in the morning rather than the evening. This finding implies two things. First, analysing data through the lens of biological time, not solely external clock time, may play a crucial role in understanding the results of chronotherapy trials. Second, this result is consistent with commonly prescribed antihypertensive regimens having maximal efficacy in the *middle* of the day versus the evening (Additional file 1: Figure S1). If antihypertensive efficacy peaked in the evening, both morning and late chronotypes would be expected to have better outcomes when dosing in the evening (Additional File 1: Figure S1B1). In contrast, an efficacy curve that peaks at midday could explain the finding that morning dosing is superior for morning chronotypes, whereas evening dosing is superior for evening chronotypes (Additional File 1: Figure S1B2). Importantly, this is a prediction based only on modelling scenarios, as no midday group was tested in TIME.

We recommend that hypertension chronotherapy trialists survey participants’ chronotypes and consider including measurements of personal biological time (e.g., DLMO, actigraphy data or validated chronotype questionnaires) as covariates in their models. Moreover, they should exclude shift workers, as the TIME trial did, or monitor their population’s relative levels of chrono-disruption through circadian tracking.

#### Treatment adherence

One commentary on the original TIME study highlighted the lack of difference in the primary outcome between a.m. and p.m. dosing; it concluded, *“From a scientific point of view*,* patients have a choice as to when to take their medication*,* but we strongly recommend taking blood pressure medication in the morning since adherence to antihypertensive medication is proven to be worse at bedtime.”* [34] This recommendation is partly based on research using smart pill bottles, which found that evening dosers missed doses approximately 1.8 times more often than morning dosers [35]. 

This statement prompts a critical question: *if adherence to medication at bedtime is known to be worse*,* and if there is no real difference between morning and evening dosing for hypertension medications*,* why were outcomes in TIME not worse in the evening dosing group than in the morning dosing group*? Differential adherence could have hidden an actual difference in outcomes between the dosing groups in TIME.

Participants in TIME were asked every three months if they were still taking all their antihypertensive medications at their assigned time, i.e., if they were adherent to their assigned dosing time. Those who responded negatively were considered “ever nonadherent” in the primary intention-to-treat analysis. Participants who reported nonadherence were asked to specify further if this was because they were taking one or more antihypertensive medications at a different dosing time or if they were not taking their medication at all. This approach to analysing participant-reported adherence to assigned dosing time over a relatively long interval did not permit the measurement of shorter periods of temporary nonadherence to dosing time, or missed doses, between follow-up points or the return to adherence after reported nonadherence. Nonetheless, we can approximate adherence using a simple linear model based on prior estimates from the literature, which capture the frequency of missed doses in various dosing schedules.

**Fig. 3:**
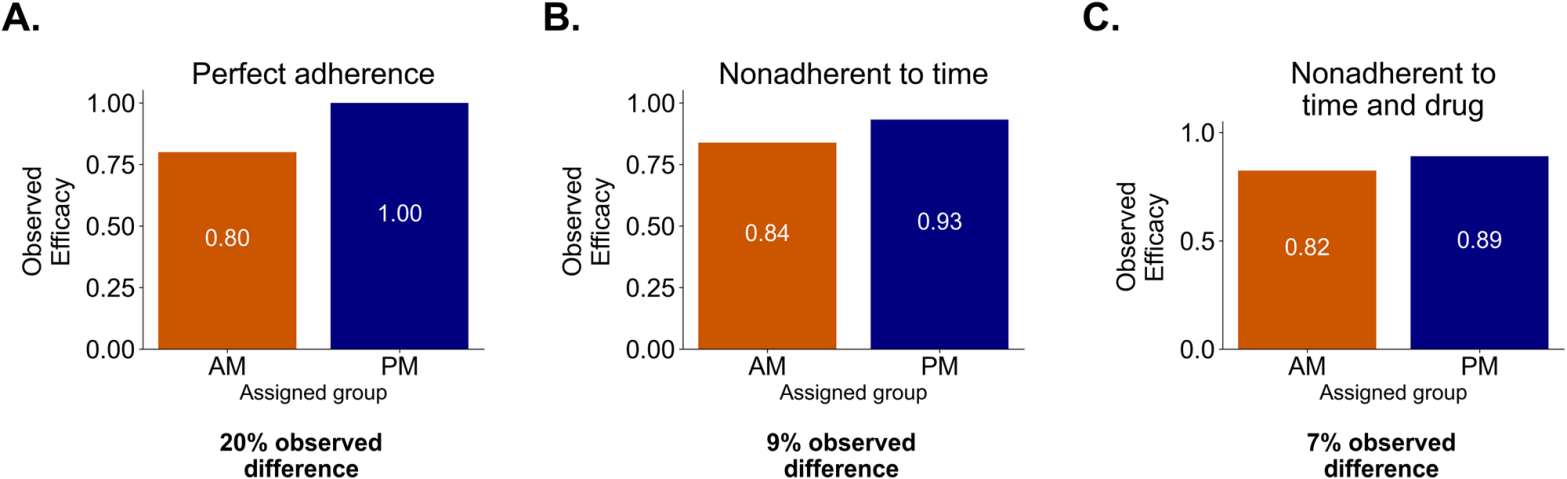
Increasing nonadherence attenuates simulated time-of-day effects. **A** Simulated efficacy under perfect adherence, assuming a true 20% advantage of p.m. over a.m. doing, such that the observed a.m. - p.m. difference matched the underlying effect. **B** Simulated efficacy when participants randomly switch dosing time at rates proportional to those observed in TIME during the first quarter of follow-up, reducing the apparent a.m. - p.m. difference (a.m. efficacy is 84%, p.m. efficacy is 93%). **C** As in (**B**), but with an additional 7% reduction in efficacy applied to p.m. dosing to reflect lower intake adherence; here, the true 20% effect appears only as a 7% difference (a.m. efficacy is 82%, p.m. efficacy is 89%).

Figure 3 illustrates how differential non-adherence can substantially blunt an underlying true effect. As in the prior dosing discussion above, this simple model omits many potentially relevant factors (e.g., the dynamics of missing doses and how these fully translate to changes in efficacy) and serves primarily to illustrate how adherence differences can obscure real differences between groups. As nonadherence increases in the p.m. group relative to the a.m. group, the observed difference between the two groups reduces. Higher differential nonadherence could reduce the observed difference to zero (or beyond, to the point where evening dosing appears worse than morning dosing due to substantially fewer doses being taken), despite a real difference between the groups under perfect adherence. These simulations, which simplify adherence dynamics reported in previous research, are intended to illustrate how time-of-day differential non-adherence could mask real chronotherapeutic benefits in pragmatic trials where adherence is not accurately measured [35].                                                                               We recommend that trials of hypertension chronotherapy measure adherence in some manner, e.g., by asking participants to regularly attest to their adherence to dosing time and intake or estimate the fraction of days with missed or wrongly-times doses, and when possible, using objective measures, such as electronic pill bottle trackers, observed administration, or mass-spectrometry-based metabolomics of urine to verify antihypertensive drugs taken (e.g. amlodipine, felodipine, nifedipine, verapamil, diltiazem, lisinopril, ramipril, perindopril, enalapril, losartan, irbesartan, candesartan, indapamide, furosemide, bendroflumethiazide, hydrochlorothiazide, spironolactone metabolite, atenolol, labetalol, bisoprolol, metoprolol, doxazosin and moxonidine) [36, 37]. 

#### Drug metabolism: pharmacokinetics and pharmacodynamics

The TIME study recruited participants taking any classes of antihypertensive drugs [10]. This pragmatic decision would have made it challenging to conduct an analysis that accounted for pharmacokinetics (PK), pharmacodynamics (PD), drug-drug interactions, and combination therapies. Evidence suggests that drugs with shorter effective half-lives tend to have more easily detectable chronotherapeutic effects [38]. 

**Fig. 4:**
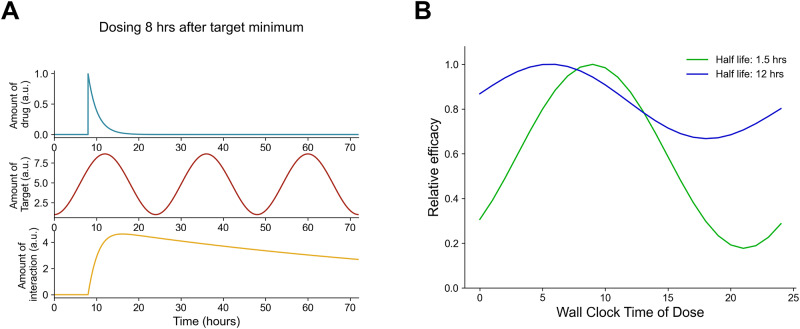
Effect of drug half-life on observable time-of-day differences in efficacy. **A** Simple differential equations model of a drug interacting with an oscillatory target. Shown is a 1.5-hour half-life dose given 8 h after the target minimum. Efficacy at that dose time is defined as the average (or cumulative) drug-target interaction over the 72-hour period of interest. **B** Relative efficacy across all dosing times (relative to target minimum) for two different half-lives (blue = 1.5 h, orange = 12 h). The shorter half-life drug shows a more pronounced best and worst dosing time (minimum efficacy < 20% of maximum), whereas the longer half-life drug shows a blunted profile (minimum efficacy is approx. 70% of maximum).

Figure 4 presents results from a simple one-compartment (i.e., representing the body as a single, well-mixed compartment) PK model in which the efficacy of a drug is influenced by circadian variation in target abundance or activity (e.g., presence of methylguanine-DNA methyltransferase interacting with temozolomide[39]; see https://www.github.com/ojwalch/time-simulations*).* In this simulation, a drug with a 1.5-hour half-life shows a clear time-of-day dependence, whereas a longer half-life drug (12 h) has a blunted efficacy profile. These simulated half-lives are consistent with those of commonly used blood pressure-lowering drugs, such as nifedipine (approximately 2 h) and lisinopril (12 h). Furthermore, circadian rhythms can modulate the PK/PD of drugs. Recent evidence suggests that the uptake and metabolism of drugs can be influenced by the time of day at which they are taken [40–42]. While this could be a driving factor behind a chronotherapeutic effect, it could also mask a time-of-day effect. For example, if an orally administered drug is poorly absorbed or metabolised at the biological time when its target is most bioavailable, the potential chronotherapeutic benefit could be negated.

Future hypertension chronotherapy trials could benefit from selecting a single drug intervention with known pharmacokinetics and pharmacodynamics and a mechanistic hypothesis for the anticipated circadian effect.

### Additional factors to consider in hypertension chronotherapy trials

#### Peripheral clocks

In our discussion so far, we have referred to a monolithic “biological time.” In fact, clocks exist throughout the body from single cells to organ systems. While the central clock in the brain responds primarily to light cues, peripheral tissue clocks dynamically update their sense of time in response to light and other factors such as meal timing and exercise. A thorough characterisation of a participant’s peripheral clocks, which may be the source of time-varying targets that enable chronotherapy, could involve wearables that track light exposure and activity levels, food diaries, and other measurement devices, such as continuous glucose and blood pressure monitors [43]. 

#### Complexity of dosing schedule

The earlier discussion contrasted external clock time dosing with biological time dosing, but did not specify how dosing according to biological time could be achieved. Past work has proposed anchoring dosing time to biological markers, such as wake time or bedtime, e.g., “take your pill after waking” [44]. Such an approach is more personalised and may better reflect a person’s circadian time. However, the ability of sleep timing to shift faster than circadian time means that there is no guarantee this will always be the case (e.g., jet lag).

In practice, drugs for which the efficacy curve has maxima and minima near typical wake and sleep times may not need dosing instructions to recommend sleep or wake times, as “morning” and “evening” would be sufficient proxies. On the other hand, interventions with efficacy curves in which the best and worst times are not readily aligned to easily identified biomarkers will require specific tailoring for a chronotherapeutic difference to be observed. For drugs where the best time is in the middle of the day, a dosing time of “seven hours after wake time” may be too challenging for patients to remember consistently, and alternative drug-delivery mechanisms, such as delayed-release capsules or automated reminders, could be more effective.

#### Circadian amplitude and profile effects

Rhythms are defined by their period (length of cycle), phase (peak time), and amplitude (magnitude of the difference between the peak and trough). Traditionally, circadian interventions, from light therapy to drug timing, have focused on phase rather than amplitude [45]. Yet data suggest marked changes in the amplitude of specific rhythms as individuals age or adopt irregular schedules (such as shift work), which could have implications for drug timing effects. For instance, consider an individual with a blunted circadian rhythm such that their daily variation in a key drug target throughout the day is minimal. There would likely be little circadian effect related to target availability for this person.

Evidence suggests that this could occur in current chronotherapy trials, specifically in the context of biological sex. Several studies have shown that women exhibit more pronounced circadian amplitudes than men [3]. At the same time, other studies have identified sex differences in time-of-day efficacy, for example, with significant time-of-day differences seen only in a female sub-cohort [46]. This by itself is not proof of amplitude as the cause of observed between-group differences. Indeed, the current evidence is limited and future work is needed to explore whether amplitude could be a possible explanatory factor for sex differences in chronotherapeutic effect. Nonetheless, these observations suggest that there could be value in controlling for any factor, such as age, sex, and shift work status, which could be expected to alter circadian amplitude. Here again, objective behavioural and metabolic measures, such as actigraphy or continuous body temperature and heart rate, would be required to capture potentially relevant information on circadian amplitudes among trial subjects.

In the specific context of hypertension trials, marked differences in the degree and direction of BP change during sleep versus awake can be observed among participants (i.e. extreme dippers, dippers, non-dippers, and risers) with significant day-to-day variability. Special populations are more likely to present unusual patterns of nighttime blood pressure, such as patients with neurological disorders due to autonomic failure, sleep disorders (e.g. obstructive sleep apnoea, restless leg syndrome, insomnia), chronic kidney disease, metabolic and endocrine disorders (e.g. obesity, type-2 diabetes, primary aldosteronism, Cushing’s syndrome) [47]. Thus, 24-hour blood ambulatory blood pressure monitoring (24-h ABPM) data collected over several days can help identify these profiles within the participant pool and illuminate what effects, if any, are observed on efficacy as a function of dosing time [48]. While there is a clear value in ABPM, the cost of repeated 24-h ABPM would have been prohibitive for a trial of TIME’s size. Indeed, BedMed only ran a small validation study within the entire trial, which was not necessarily representative of the whole study population. With this caveat, it showed that bedtime dosing was associated with better nighttime blood pressure control. Therefore, the choice of whether or not to include 24-h ABPM in future trials should be determined both by practicality and the specific research questions under consideration. In particular, research should focus on validated wearable devices capable of measuring continuous blood pressure throughout 24 h, including ‘asleep’ periods [47]. Additional file 1 contains Table S1 which summarises the advantages and disadvantages of various data collection options for controlling relevant factors in chronotherapy trials.

### Key insights for hypertension chronotherapy trials

Chronotherapy has been proposed as a means to patient outcomes; however, while some hypertension chronotherapy trials have yielded positive results, the inconsistent replication of these findings across studies remains a challenge to implementation. This variability may be partly attributed to differences in the study design and data collection methods. As such, standardising research protocols and conducting larger trials may help clarify the potential of chronotherapy in treating hypertension, as well as its application across other medical conditions.

Using the TIME study as a case example, we highlighted a few factors relevant to hypertension chronotherapy trials. Using mathematical simulations, we demonstrated how differences in population characteristics, such as prior medication experience and diversity in individual biological time, could influence the detection of chronotherapeutic effects. We argued that these factors should be considered when designing, comparing, and interpreting trials. Furthermore, chronotherapeutic interventions must be clearly defined, considering the potential variations between external clock time, sleep-wake behaviour, and circadian rhythms. Attention may even be given to a dynamic circadian rhythm-marker-based intervention, with participants guided on when to take their medication daily according to the results of circadian monitoring. Adherence is crucial in all clinical trials, and accurately capturing the timing and occurrence of actual drug administration may be key to interpreting the results of chronotherapeutic trials. Moreover, time-dependent nonadherence (where individuals are less likely to be adherent at specific times of day) is likely to influence the observed efficacy of chronotherapeutic interventions, and optimal implementation of an effective chronotherapy intervention in clinical care may require different timing recommendations for patients based on their likelihood of adherence. Finally, drug metabolism is an additional factor to control in trials of chronotherapy, where interaction with time-varying targets and longer half-lives can potentially blunt time-of-day effects and mask differences between groups.

Our recommendations are based on the experience of running a large hypertension clinical trial with time-of-day dosing intervention. As with all mathematical modelling, the presented outputs of the simulations depend entirely on the assumptions made based on previous evidence from research in circadian biology. Simulation research can shed light on the potential effects of trial design decisions, and publication will permit shared learning.

Fully accounting for circadian timing in hypertension trials would entail navigating the vast complexity of circadian rhythms, including central versus peripheral oscillators, as well as factors such as metabolism, meal timing, physical activity, and numerous interaction effects. An exhaustive list of factors that could be relevant to chronotherapy trials is daunting. A pragmatic solution may be to use simple questionnaires to capture potentially important participant-reported data. For example, participants may be asked to report how long they have been on their previous drug regimen, to report their level of adherence regularly, and to identify their natural sleep-wake preferences (i.e., individual chronotype). Moreover, technological solutions such as activity trackers (including those built into smartphones), wearable devices, simple urine tests using mass spectrometry-based metabolomics, and smart pill bottles capable of tracking adherence are increasingly making it significantly easier to capture previously elusive variables. These innovations have the potential to greatly enhance the quality and depth of data in future hypertension chronotherapy trials, thereby providing researchers with more reliable results. However, their use must be balanced against the risk of overburdening trial participants.

However, it must be remembered that a clinical trial and the interventions they test must be both scientifically sound and practically feasible. The ultimate objective is to formulate chronotherapy approaches that are reasonably robust to the considerations identified here, are feasible in healthcare contexts, and are acceptable to patients.

## Conclusions

We recommend careful consideration of study design choices to ensure that the necessary data are collected to evaluate the effect of chronotherapeutic interventions in hypertension. While there will never be a perfect clinical trial, the barriers to tracking some of the most crucial factors are lower than ever. By making small to moderate adjustments to future clinical trials evaluating chronotherapeutic effects, researchers can increase the likelihood that their work will lead to clinically meaningful insights.

## Supplementary Information

Below is the link to the electronic supplementary material.

Supplementary Material 1: Figure S1 and Table S1. Figure S1 - Effect of efficacy-peak timing on optimal dosing by chronotype. Table S1 - Tools available to researchers to collect chronotherapy relevant data.

## Data Availability

Data sharing is not applicable to this article as no datasets were generated or analysed during the current study. All code to generate the figures is available on GitHub (https://www.github.com/ojwalch/time-simulations).

## Abbreviations

BP: blood pressure
DLMO: dim light melatonin onset
PD: pharmacodynamics
PK: pharmacokinetics
RCTs: randomised clinical trials
SCN: suprachiasmatic nucleus
24-h ABPM: 24-hour ambulatory blood pressure monitoring
