# Supplementary material for: Design considerations for hypertension chronotherapy trials: insights from experience and modelling
Source: BMC Med. 2026 May 19;24:391. doi: 10.1186/s12916-026-04915-8 (PMC13352859; doi:10.1186/s12916-026-04915-8)
Supplement: Supplementary file 1 — Supplementary Material 1: Figure S1 and Table S1. Figure S1 - Effect of efficacy-peak timing on optimal dosing by chronotype. Table S1 - Tools available to researchers to collect chronotherapy relevant data. [file 12916_2026_4915_MOESM1_ESM.docx]

# Additional file 1

**
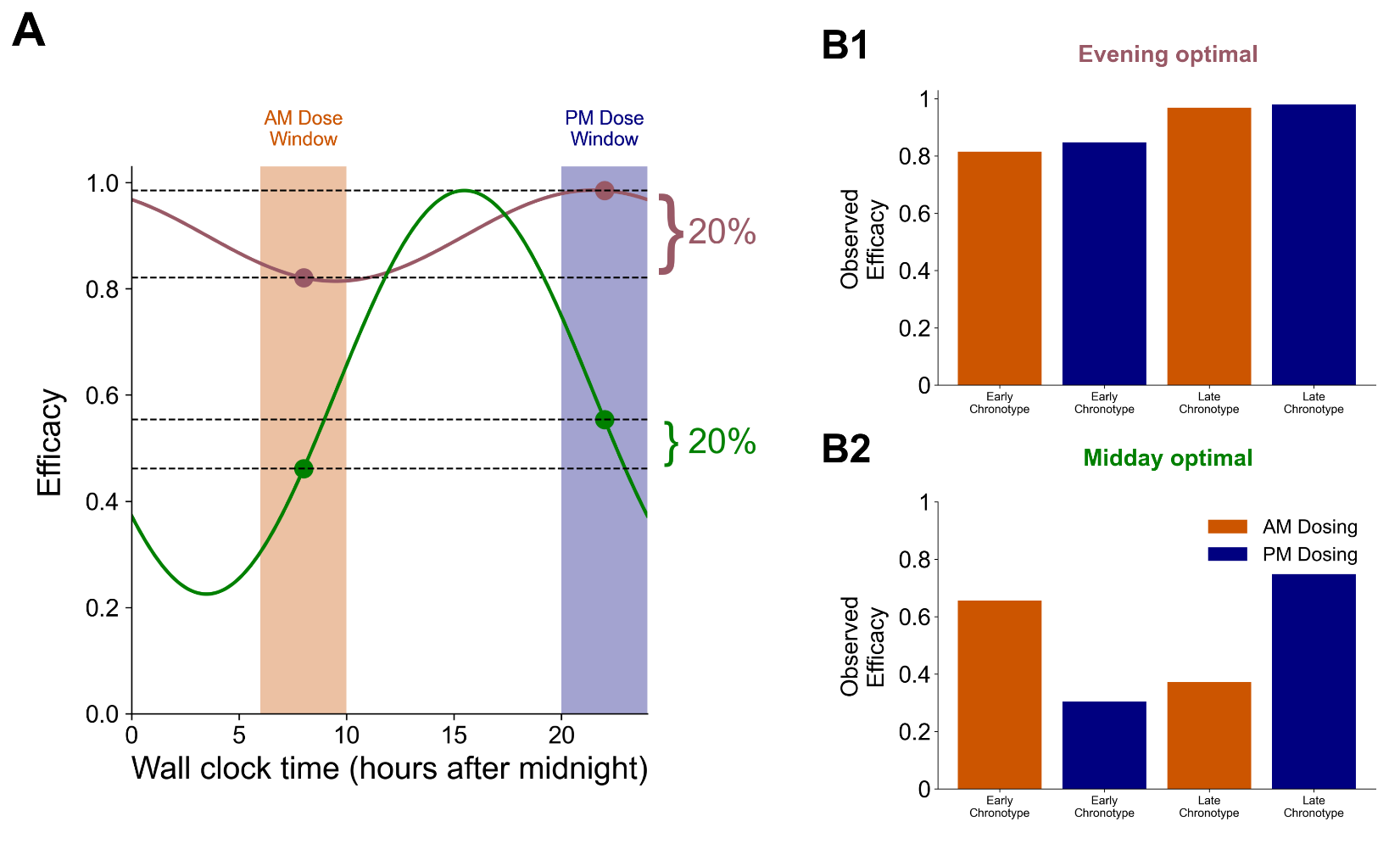
**

**Figure S1. Effect of efficacy-peak timing on optimal dosing by chronotype.** (A) Two hypothetical efficacy curves with an observable 20% advantage of p.m. over a.m. dosing. The magenta curve is the homogenous population with evening optimal dosing from Figure 2. The green curve also has 20% higher efficacy with p.m. versus a.m. dosing, but peaks at midday rather than in the evening, where p.m. is 20% better than a.m., but the true peak is in the middle of the day. (B1,B2) Simulated a.m. and p.m. dosing outcomes for early and late chronotypes, assuming late types are 2 hours delayed and early types are 2 hours advanced relative to the plotted curves. When evening is the true optimal time (A, magenta), p.m. dosing is slightly more effective for both early and late types (B1). When midday is optimal (A, green), early types do better with a.m. dosing and late types with p.m. dosing (B2). The pattern observed in the TIME chronotype sub-study is more consistent with a midday-optimal efficacy curve.(16)

#

| **Tool** | **Advantages** | **Disadvantages** |
| --- | --- | --- |
| Surveys and questionnaires (e.g., chronotype questionnaires, food sleep surveys, etc.) | - Affordable - Low burden | - Recall bias - Pre/post surveys unable to capture day-to-day variations in circadian rhythms - Differences between sleep/wake habits and “circadian time” |
| Research-grade actigraphy with and without light tracking (e.g., ActiGraph, Condor, GENActiv) | - Standard in the field - Large body of prior research | - Expense - Can be challenging to have research participants wear reliably - Unreliability of measured light sensor data |
| Consumer-grade wearable devices (e.g., Apple Watch, Fitbit) | - More affordable than research-grade actigraphy - Participants may already own one - Can be easier to have participants reliably wear them | - Expense (still higher than surveys) - Significant diversity of devices and reporting units - Black box algorithms - Battery life - Lack of light sensor data |
| Disease-relevant devices (e.g., continuous glucose monitors, blood pressure monitors) | - High-resolution tracking of peripheral clock-related measures - More real-time measures of desired outputs | - Expense - Lack of prior consensus on how to extract circadian parameters from the data |
| Smartphone apps | - Hybrid option, potentially combining consumer-grade wearable devices, surveys, and other forms of data capture (e.g., photographic documentation of pill adherence and/or meals) | - Would require custom build to match study specifics - All disadvantages of component elements, such as consumer-grade wearable devices, apply |

#### **Table S1. Table summarising tools available to researchers to collect chronotherapy-relevant data**
